# *RFC1* in an Australasian neurological disease cohort: extending the genetic heterogeneity and implications for diagnostics

**DOI:** 10.1093/braincomms/fcad208

**Published:** 2023-07-25

**Authors:** Carolin K Scriba, Igor Stevanovski, Sanjog R Chintalaphani, Hasindu Gamaarachchi, Roula Ghaoui, Darshan Ghia, Robert D Henderson, Nerissa Jordan, Antony Winkel, Phillipa J Lamont, Miriam J Rodrigues, Richard H Roxburgh, Ben Weisburd, Nigel G Laing, Ira W Deveson, Mark R Davis, Gianina Ravenscroft

**Affiliations:** Rare Genetic Diseases and Functional Genomics Group, Centre for Medical Research, University of Western Australia, Harry Perkins Institute of Medical Research, QEII Medical Centre, Nedlands, WA 6009, Australia; Neurogenetics Laboratory, Department of Diagnostic Genomics, PP Block, QEII Medical Centre, Nedlands, WA 6009, Australia; Genomics Pillar, Garvan Institute of Medical Research, Sydney, NSW 2010, Australia; Centre for Population Genomics, Garvan Institute of Medical Research and Murdoch Children’s Research Institute, Sydney, NSW 2010, Australia; Genomics Pillar, Garvan Institute of Medical Research, Sydney, NSW 2010, Australia; Centre for Population Genomics, Garvan Institute of Medical Research and Murdoch Children’s Research Institute, Sydney, NSW 2010, Australia; School of Clinical Medicine, Faculty of Medicine and Health, University of New South Wales, Sydney, NSW 2050, Australia; Genomics Pillar, Garvan Institute of Medical Research, Sydney, NSW 2010, Australia; Centre for Population Genomics, Garvan Institute of Medical Research and Murdoch Children’s Research Institute, Sydney, NSW 2010, Australia; School of Computer Science and Engineering, University of New South Wales, Sydney, NSW 2052, Australia; Department of Neurology, Royal Adelaide Hospital, Adelaide, SA 5000, Australia; Adelaide Medical School, Faculty of Health and Medical Sciences, University of Adelaide, Adelaide, SA 5000, Australia; UWA Medical School, University of Western Australia, Perth, WA 6009, Australia; Neurology and Stroke Unit, Fiona Stanley Hospital, Murdoch, WA 6150, Australia; Centre for Clinical Research, University of Queensland, Herston, QLD 4006, Australia; Department of Neurology, Fiona Stanley Hospital, Perth, WA 6150, Australia; Department of Neurosciences, Griffith University, Sunshine Coast University Hospital, Mount Gravatt, QLD 4111, Australia; Neurogenetic Unit, Royal Perth Hospital, Perth, WA 6000, Australia; Neurology Department, Auckland City Hospital, Auckland, New Zealand; Centre for Brain Research Neurogenetics Research Clinic, University of Auckland, Auckland, New Zealand; Program in Medical and Population Genetics, Broad Institute of MIT and Harvard, Cambridge, MA, USA; Preventive Genetics Group, Centre for Medical Research, University of Western Australia, Harry Perkins Institute of Medical Research, QEII Medical Centre, Nedlands, WA 6009, Australia; Genomics Pillar, Garvan Institute of Medical Research, Sydney, NSW 2010, Australia; Centre for Population Genomics, Garvan Institute of Medical Research and Murdoch Children’s Research Institute, Sydney, NSW 2010, Australia; School of Clinical Medicine, Faculty of Medicine and Health, University of New South Wales, Sydney, NSW 2050, Australia; Neurogenetics Laboratory, Department of Diagnostic Genomics, PP Block, QEII Medical Centre, Nedlands, WA 6009, Australia; Rare Genetic Diseases and Functional Genomics Group, Centre for Medical Research, University of Western Australia, Harry Perkins Institute of Medical Research, QEII Medical Centre, Nedlands, WA 6009, Australia

**Keywords:** ataxia, CANVAS, *RFC1*, sensory neuropathy, STR

## Abstract

Cerebellar ataxia, neuropathy and vestibular areflexia syndrome is a progressive, generally late-onset, neurological disorder associated with biallelic pentanucleotide expansions in Intron 2 of the *RFC1* gene. The locus exhibits substantial genetic variability, with multiple pathogenic and benign pentanucleotide repeat alleles previously identified. To determine the contribution of pathogenic *RFC1* expansions to neurological disease within an Australasian cohort and further investigate the heterogeneity exhibited at the locus, a combination of flanking and repeat-primed PCR was used to screen a cohort of 242 Australasian patients with neurological disease. Patients whose data indicated large gaps within expanded alleles following repeat-primed PCR, underwent targeted long-read sequencing to identify novel repeat motifs at the locus. To increase diagnostic yield, additional probes at the *RFC1* repeat region were incorporated into the PathWest diagnostic laboratory targeted neurological disease gene panel to enable first-pass screening of the locus for all samples tested on the panel. Within the Australasian cohort, we detected known pathogenic biallelic expansions in 15.3% (*n* = 37) of patients. Thirty indicated biallelic AAGGG expansions, two had biallelic ‘Māori alleles’ [(AAAGG)_exp_(AAGGG)_exp_], two samples were compound heterozygous for the Māori allele and an AAGGG expansion, two samples had biallelic ACAGG expansions and one sample was compound heterozygous for the ACAGG and AAGGG expansions. Forty-five samples tested indicated the presence of biallelic expansions not known to be pathogenic. A large proportion (84%) showed complex interrupted patterns following repeat-primed PCR, suggesting that these expansions are likely to be comprised of more than one repeat motif, including previously unknown repeats. Using targeted long-read sequencing, we identified three novel repeat motifs in expanded alleles. Here, we also show that short-read sequencing can be used to reliably screen for the presence or absence of biallelic *RFC1* expansions in all samples tested using the PathWest targeted neurological disease gene panel. Our results show that *RFC1* pathogenic expansions make a substantial contribution to neurological disease in the Australasian population and further extend the heterogeneity of the locus. To accommodate the increased complexity, we outline a multi-step workflow utilizing both targeted short- and long-read sequencing to achieve a definitive genotype and provide accurate diagnoses for patients.

## Teaser

Scriba *et al.* report that pathogenic *RFC1* expansions contribute substantially to disease within Australasia. A workflow is suggested that integrates short- and long-read sequencing to facilitate accurate diagnosis and overcomes many challenges associated with *RFC1* heterogeneity. The prevalence of complex alleles is highlighted, including the identification of three novel repeat motifs.

## Graphical Abstract

**Graphical Abstract:**
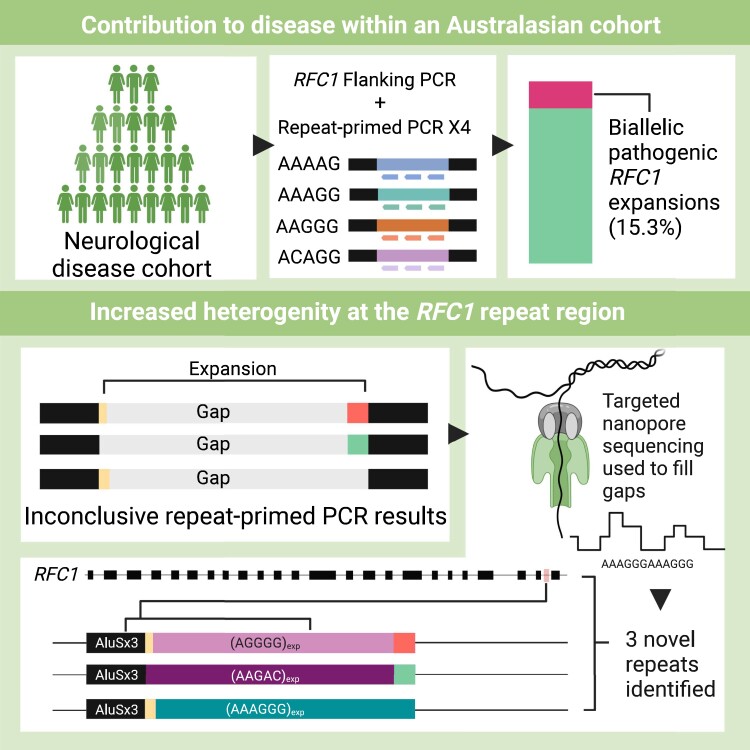

## Introduction

Cerebellar ataxia, neuropathy and vestibular areflexia syndrome (CANVAS; Online Mendelian Inheritance in Man: 614575) is an autosomal recessive, slowly progressive disease characterized by a triad of features: sensory neuropathy/neuronopathy, cerebellar dysfunction and bilateral vestibulopathy.^1‐3^ First described as a distinct syndrome by Szmulewicz *et al.*,^2^ the genetic basis for the disease was independently discovered in 2019 by both Cortese *et al.*^4^ and Rafehi *et al.*^5^ CANVAS is associated with biallelic pathogenic pentanucleotide expansions within the second intron of the *RFC1* gene. In affected patients, the pathogenic allele (AAGGG)_exp_ differs from the reference allele (AAAAG)_11_ in terms of both size and composition.^4,5^ In addition, the study identified the presence of two alternate benign expansions at the locus, (AAAAG)_exp_ and (AAAGG)_exp_.^4^

Since the initial observation, understanding of the heterogeneity of the locus has continued to grow, with reports of multiple, additional alleles observed within different populations. (AAGAG)_exp_ and (AGAGG)_exp_ were observed within a Canadian and Brazilian cohort, although the pathogenicity of these repeat motifs has not yet been determined.^6^ An additional pathogenic repeat motif, (ACAGG)_exp_, was discovered within individuals from Indonesia, Niue and Japan. Another allele discovered within the Māori populations of New Zealand and the Cook Islands has also been associated with disease. Often described as the Māori allele, it is a conjugation of two previously described repeat motifs [(AAAGG)_15–25_(AAGGG)_exp_ (AAAGG)_10_].^7‐10^ Recently, two papers suggested that single-nucleotide variants (SNVs) in trans with pathological expansions can also cause disease.^11,12^ However, this paper specifically addresses the issue of heterogeneity in pathological repeat expansions in patients with biallelic expansions.

European population cohort studies have demonstrated that pathogenic *RFC1* expansions underlie a substantial proportion of clinically suspected CANVAS as well as ataxia and neuropathy cases.^4,13‐15^ It has been postulated that the disease shows spatial progression initially involving sensory neurons, with cerebellar and vestibular dysfunction manifesting later.^13^ Thus, *RFC1* should still be considered in cases not presenting with all three features characteristic of the disease. There is emerging evidence that, in rare cases, *RFC1* expansions may also underlie a range of other phenotypes, including behavioural-psychiatric symptoms, multi-systemic neurodegeneration and Parkinsonian features.^16‐20^

So far, most studies have relied on repeat-primed PCR (RP-PCR) to identify pathogenic *RFC1* expansions. Although this is the standard diagnostic practice for tandem repeat disorders, the method is limited to known repeat motifs and can only provide an insight into the first hundred repeats of the expansion. This poses substantial challenges for accurate genetic diagnosis of patients with *RFC1* expansions due to the complex genetics and large size of pathogenic expansions.

The emergence of long-read sequencing technologies provides a platform for a more comprehensive investigation of the *RFC1* locus. Long-read sequencing technologies from Pacific Biosciences and Oxford Nanopore Technologies (ONT) are able to generate continuous sequences ranging from 10 kb to several megabases from a single molecule and have the capacity to phase alleles.^21‐23^ Methods to target the *RFC1* repeat locus specifically have been successful using both CRISPR-Cas9 and computational targeting with ONT’s ReadUntil function.^24,25^ These techniques enable targeted capture of the entire repeat expansion in a single continuous sequence, making long-read sequencing a powerful tool for the investigation of complex and heterogeneous short tandem repeat (STR) loci.

By screening 242 patients with neurological disease, we show that pathogenic *RFC1* expansions underline a substantial proportion of ataxia and neuropathy patients within the Australasian population. Through a combination of standard PCR methods and targeted long-read sequencing, we also describe several novel repeat motifs that have not been previously reported, demonstrating further complexity of expanded alleles at this locus. The increased genetic heterogeneity emphasizes the need for updated diagnostic methods with regard to *RFC1-*related disease. Thus, we outline a streamlined diagnostic protocol for the accurate detection of *RFC1* repeat expansions utilizing both short- and long-read sequencing to provide patients with definitive genetic diagnoses.

## Materials and methods

### Cohort

This project was approved by the Human Research Ethics Committee of the University of Western Australia (RA/4/20/1008). The cohort (*n* = 242, 117 females, 125 males) was recruited from patients who remained undiagnosed following testing on the PathWest Laboratory Medicine neurogenetic diagnostic gene panel (analysed on the ataxia and/or neuropathy phenotypic subpanels for SNVs and/or copy number variants within known disease genes)^26^ and patients with clinically suspected CANVAS at the Department of Diagnostic Genomics (PathWest). Repeat expansion disorders were not excluded prior to CANVAS testing. Patients (>30 years of age) with clinically suspected CANVAS (*n* = 42), ataxia (*n* = 105), neuropathy (*n* = 50) and ataxia in combination with neuropathy (*n* = 45) were included. Clinical groupings were based on clinical information provided by the requesting clinician at the time of the initial request for genetic diagnostic testing.

DNA samples from whole blood were obtained from the Department of Diagnostic Genomics (PathWest) for each of the probands. Clinical data were collected by the referring clinicians, and no further phenotyping was undertaken.

### Flanking PCR

The flanking PCR was adapted from Cortese *et al.*^4^ and was used as a screening protocol to exclude patients who did not have biallelic expansions within the *RFC1* repeat region. Flanking PCR was performed on DNA from all patients included in the study, according to the primers and specifications listed in Supplementary Table 1. Following amplification, 1 µl of PCR product was combined with 11.825 µl of Hi-Di Formamide and 0.125 µl of LIZ600 ladder and underwent fragment length separation analysis as performed by PathWest, Department of Diagnostic Genomics using the 3730 DNA analyser (Applied Biosystems). Fragment length separation data were analysed using GeneMapper software (Version 4.1). Samples with at least one peak between 340 and 370 bp (∼8–14 repeats) were excluded from further testing. Samples that did not show any peaks following GeneScan analysis were suspected to carry biallelic repeat expansions and were subsequently tested by RP-PCR.

### Repeat-primed PCR

RP-PCR was used to determine the configuration of the expansions within patient samples, based on methods described by Cortese *et al*.^4^ and Scriba *et al.*^7^ Four separate RP-PCR reactions were performed on each sample that was determined to contain a biallelic expansion by flanking PCR, to determine the repeat motif present within each sample. Primer sequences, and reaction and thermocycling conditions are provided in Supplementary Table 1. Following amplification, samples were prepared for fragment length separation analysis by combining 1 µl of PCR product with 11.875 µl of Hi-Di Formamide and 0.125 µl of LIZ1200 ladder. Fragment length separation was performed by PathWest, and the data were analysed using GeneMapper software (Version 4.1).

Following the discovery of novel repeat motifs by long-read sequencing, additional repeat-specific RP-PCRs were designed for AACAG and AAAGGG. Validation of long-read sequencing results and the prevalence of each individual novel motif within the cohort were determined by testing all patient samples that indicated a biallelic expansion following flanking PCR (*n* = 82), on each of the additional RP-PCRs. Primer sequences, and reaction and thermocycling conditions are provided in Supplementary Table 1.

### Targeted long-read sequencing

Six samples underwent computationally targeted long-read sequencing for the *RFC1* expansion. Sequencing and analysis methods have been described by Stevanovski *et al.*^24^

### Screening for biallelic expansions using next generation sequencing gene panels

The PathWest neurological disease gene panel (Version 6) now consists of 496 genes associated with neurological disease. The original panel design and validation is described in Beecroft *et al*.^26^ To capture the repeat region within the second intron of the *RFC1* gene, additional probes spanning the region were added to Version 6 of the PathWest neurological disease targeted gene panel. Probe 1 covered the region chr4: 39348349–39348469. Probe 2 covered the region chr4: 39348370–39348490 (GRCh38/hg38).

A subset of 42 patients from the cohort were tested on Version 6 of the neurological disease gene panel. Following analysis for sequence variants in known disease-associated genes, to exclude alternate genetic causes, the binary alignment map (BAM) files produced for all 42 samples were visually inspected at the *RFC1* repeat locus (Chr:39348425–39348485, GRCh38/hg38) within the integrative genomics viewer (IGV) with soft-clipping disabled. Standard CANVAS testing (flanking PCR and RP-PCR) was conducted on the samples to validate the findings from next generation sequencing (NGS).

### Analysis of 19 241 genomes

To determine the prevalence of the novel motifs identified in our cohort within the wider population and to search for additional repeat motifs at the *RFC1* locus 19 241 ethnically diverse samples from gnomAD v3^27^ were analysed. Compressed reference-oriented alignment map files from 19 241 gnomAD v3^27^ genomes were analysed using the script ‘call_non_ref_pathogenic_repeats.py’ from the package str-analysis 0.9 (https://github.com/broadinstitute/str-analysis). The pipeline first determined the different motifs present at the *RFC1* locus; next, ExpansionHunter^28^ was run for each motif to estimate the allele size. REViewer^29^ was used to generate read visualizations.

## Results

### Contribution of pathogenic *RFC1* expansions to disease

**Figure 1:**
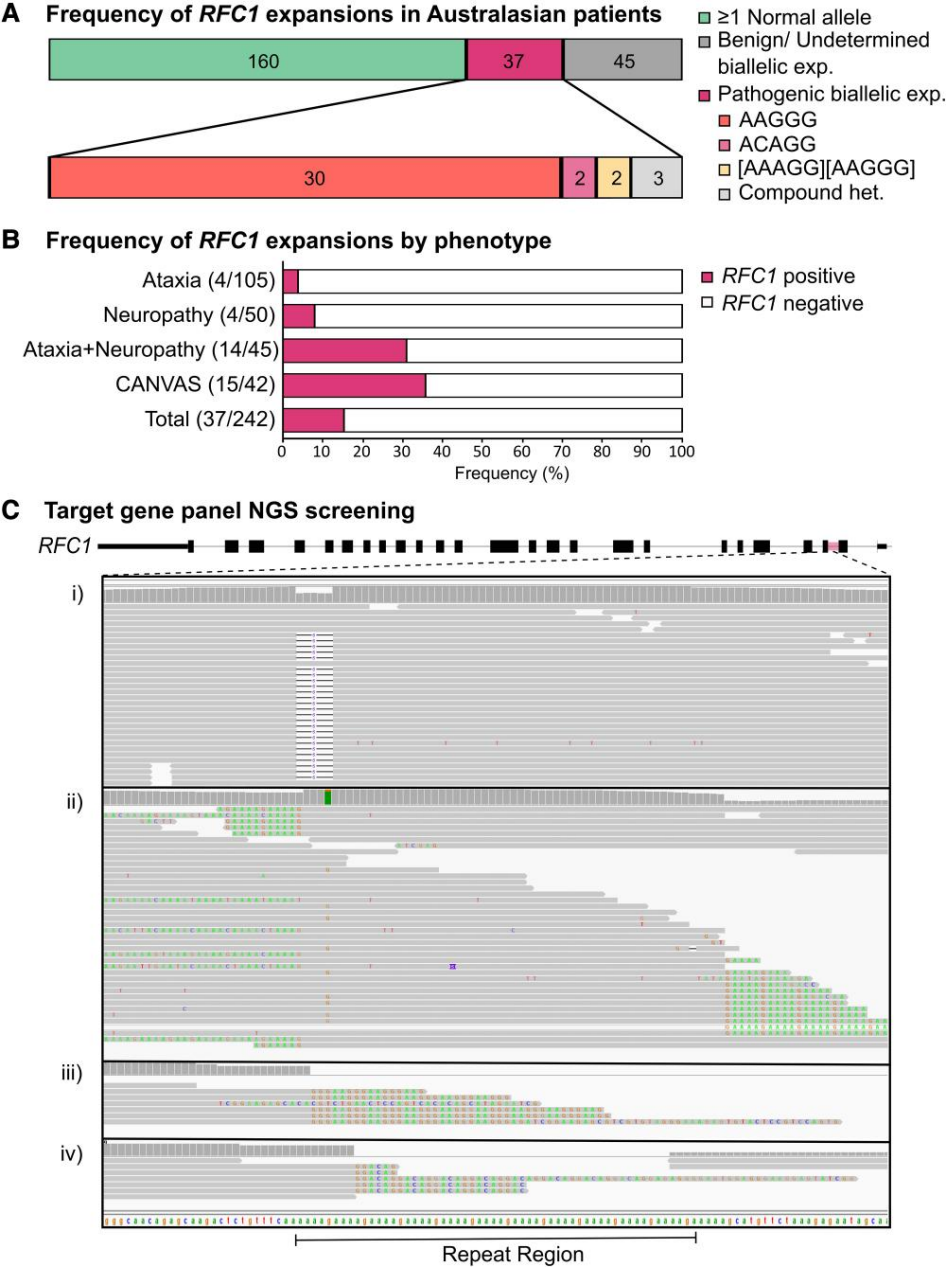
**Screening for biallelic *RFC1* expansion within Australasian, neurological disease patients.** (**A**) The figure shows the entire cohort (*n* = 242), the proportion to test positive for biallelic pathogenic expansions, and displays the breakdown of the different pathogenic alleles present (numbers represent individual patients). (**B**) Frequency of biallelic pathogenic *RFC1* expansions split by phenotypic subgroup. (**C**) Visualization of reads from the NGS-targeted gene panel in IGV aligned to the repeat region of *RFC1*. Results from four different patients are displayed to show how different genotypes align: (i) data from HP220214 show normal coverage across the repeat region with two reference alleles present; (ii) data from HP220212 show a drop in coverage directly flanking the repeat region and indicate the presence of two expanded AAAAG alleles; (iii) data from HP220201 show a drop in coverage across the repeat region and indicate the presence of two expanded AAGGG alleles and (iv) data from HP220234 show a drop in coverage across the repeat region and indicate the presence of two expanded ACAGG alleles.

Within a cohort of 242 Australasian, neurological disease patients (mean age of patients included in the study was 67 ± 11.5 years, ranging from 33 to 92 years), we identified pathogenic biallelic expansions in 15.3% (*n* = 37) of cases (Fig. 1A).

In two unrelated patients, an expanded AAGGG allele was seen in combination with novel complex alleles following testing by RP-PCR. In both cases, the majority of the second complex allele consisted of a known pathogenic motif, with only a small proportion of a second motif present near the 5ʹ end of the expansion, similar in pattern to that of the Māori allele. For the purpose of categorizing pathogenic alleles, these have been counted as the motif constituting the majority of the expansion as indicated by RP-PCR. Further discussion of complex alleles follows in a separate section of the results.

Thirty patient samples indicated biallelic expansions of the AAGGG motif, two samples had biallelic Māori alleles [(AAAGG)_exp_(AAGGG)_exp_], two samples had biallelic expansions of the ACAGG motif, two samples were compound heterozygous for the AAGGG expanded allele and the Māori expanded allele, and one sample was compound heterozygous for the AAGGG expanded allele and the ACAGG expanded allele.

For this study, patients were grouped into four phenotypic subtypes: ataxia (*n* = 105), neuropathy (*n* = 50), ataxia in conjunction with neuropathy (*n* = 45) and clinically suspected CANVAS (*n* = 42). The results for each phenotypic cohort are summarized in Fig. 1B. Within the ataxia cohort, 4.7% (*n* = 5) had biallelic pathogenic expansions. Within the neuropathy cohort, 8% (*n* = 4) had biallelic pathogenic expansions. In the cohort of patients with a combination of ataxia and neuropathy, 31.1% (*n* = 14) showed biallelic pathogenic expansions. Within the suspected CANVAS cohort, 35.7% (*n* = 15) had biallelic pathogenic expansions.

### NGS panels as a screening tool for *RFC1* expansions

Given the reasonably high prevalence of *RFC1-*related disease in patients initially tested using the ataxia and neuropathy subpanels of the targeted neurological disease gene panel at PathWest, we wanted to investigate whether expansions at the *RFC1* locus could be screened for using targeted next generation gene panels in order to streamline the diagnostic process. Additional probes flanking the intronic repeat region of *RFC1* were added to Version 6 of the gene panel.

A subset of 42 samples from the cohort were screened on the targeted gene panel that included the *RFC1* probes and by standard testing for comparison of results. Our results show that the data obtained from NGS are sufficient to screen for biallelic expansions at the *RFC1* locus. Visual inspection of the BAM files showed reads spanning the *RFC1* repeat locus for samples harbouring one or two reference alleles, with clear drops in coverage seen for samples harbouring biallelic expansions (Fig. 1C). As demonstrated in Fig. 1C, reads that span the expanded region contain multiple repeats. This provides an indication of the repeat motif present. In some cases harbouring biallelic expansions of pathogenic motifs, coverage is so low that no flanking reads are present to indicate the repeat motif present. The BAM files, when visualized in IGV, clearly show the presence or absence of the reference allele. This allows for exclusion of CANVAS when present or elicits further testing by RP-PCR or long-read sequencing if absent (Fig. 1C).

The NGS approach showed 100% concordance in identifying the presence or absence of biallelic expansions when compared with the results of standard flanking PCR and RP-PCR in 42 patients. Therefore, this method enables all patient samples tested using Version 6 of the PathWest neurological disease gene panel to be pre-screened for biallelic *RFC1* expansions.

### Complex *RFC1* repeat expansion alleles are common

**Figure 2:**
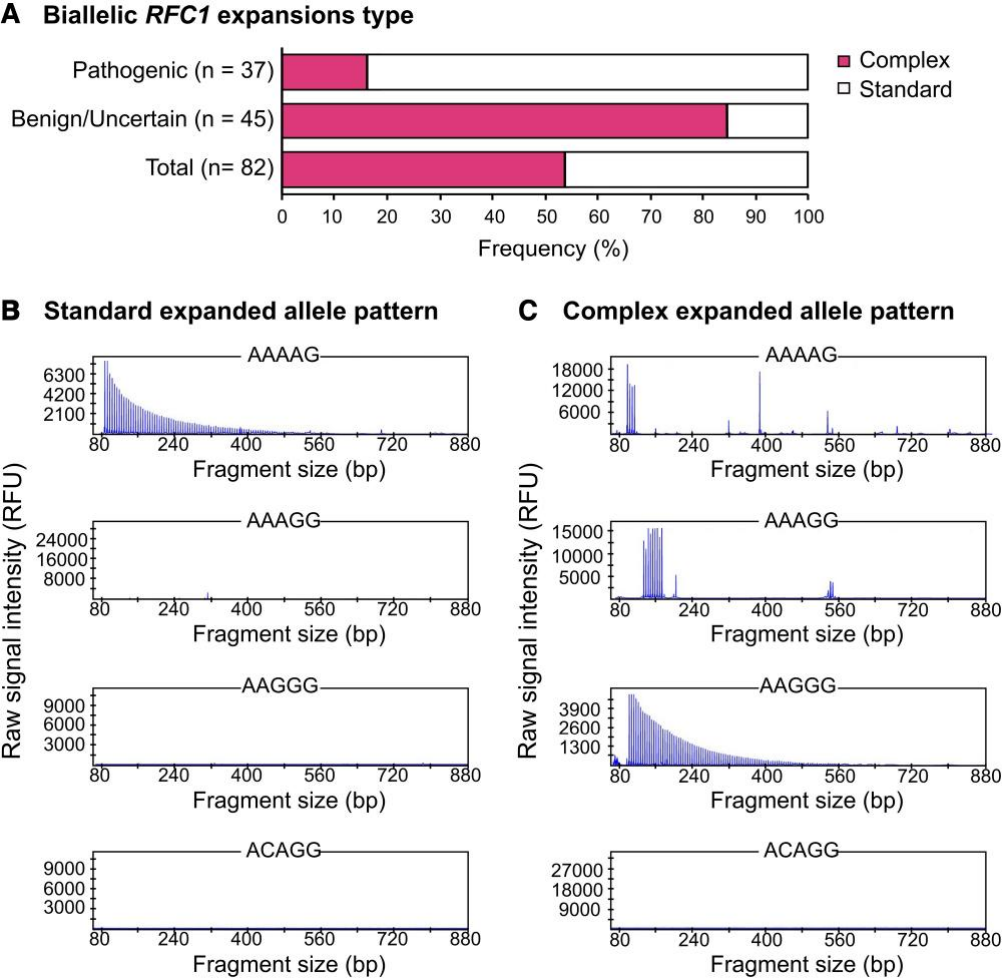
**Increased complexity of expanded *RFC1* alleles.** (**A**) Proportion of patients harbouring biallelic expansions with at least one complex allele (containing more than one repeat motif), as determined by RP-PCR in 82 patients. (**B**) Representative standard RP-PCR traces demonstrating the characteristic laddering pattern indicating the presence of a pure expanded AAAAG allele. (**C**) Representative complex allele RP-PCR traces. Blocks of peaks across multiple different RP-PCR assays indicate the presence of multiple repeat motifs constituting a single expanded allele, large gaps within inferred alleles indicate possible novel motifs.

Of the 242 samples screened by flanking PCR, 82 did not produce a PCR product, indicating the presence of biallelic expansions. These samples were then tested using RP-PCR to determine whether the expansions were pathogenic or benign. Of the 82 samples, 53.7% (*n* = 44) gave uncharacteristic laddering patterns following RP-PCR, indicating the presence of complex alleles made up of more than a single repeat motif (Fig. 2).

In 27 of these samples, there were gaps in the RP-PCR data, which signifies the presence of repeat motifs different from those being tested (Fig. 2). Samples with large gaps were then tested further using targeted long-read sequencing (where sufficient DNA was available). Three novel repeat motifs were identified: two pentanucleotide repeats: AAGAC, AGGGG, and one hexanucleotide repeat AAAGGG. These novel repeats were seen in combination with other novel and known repeat motifs.

**Figure 3:**
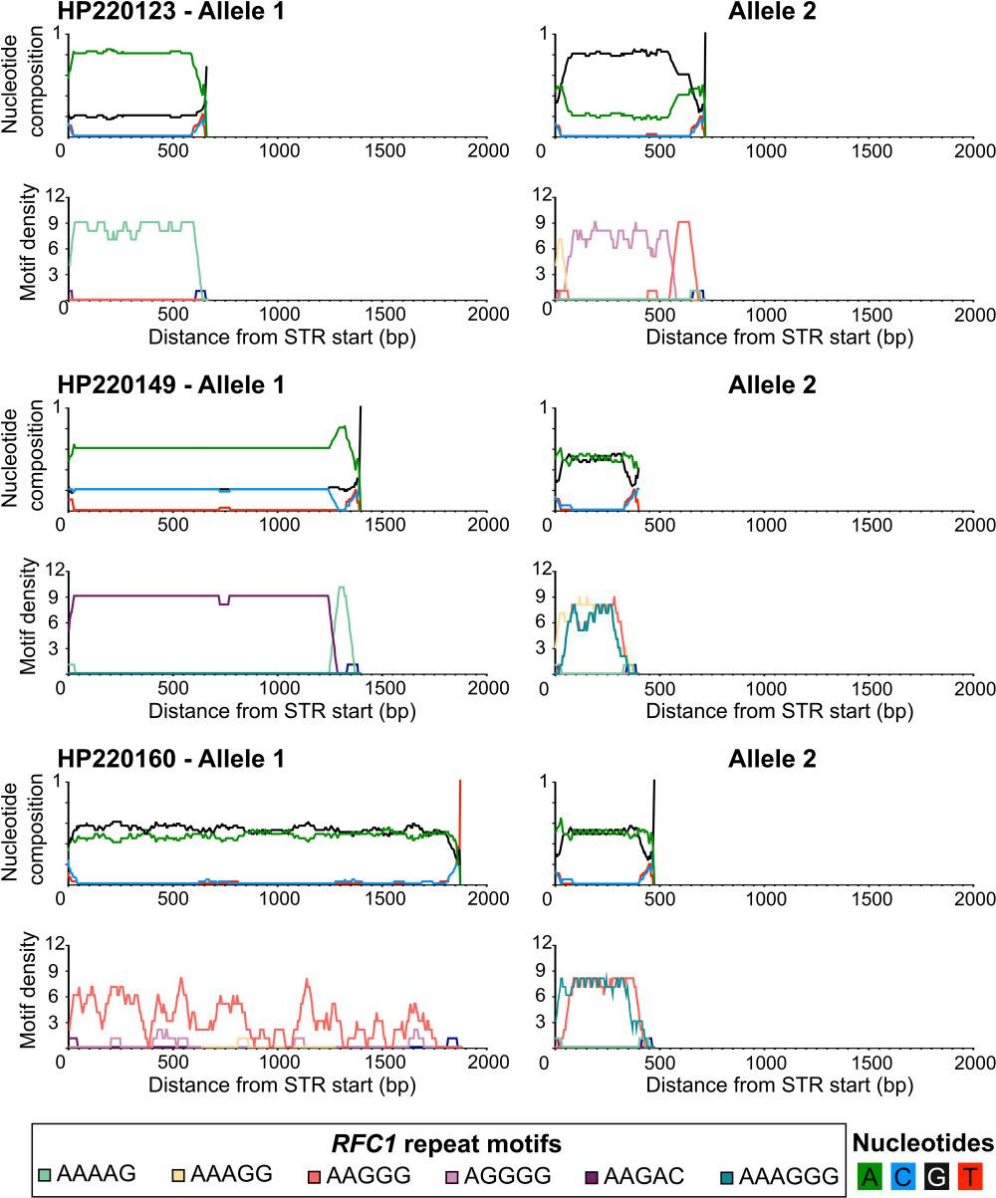
**Long-read sequencing of samples harbouring unknown expansion.** Line plots of the nucleotide composition (top) and repeat motif density (bottom) at the *RFC1* disease locus for each haplotype of three patients’ samples that underwent long-read sequencing. For example, Allele 1 of patient sample HP220149 is shown to contain 60% A nucleotides, 20% G nucleotides and 20% C nucleotides (consistent with the repeat motif AAGAC) for ∼1250 bp, the nucleotide composition then changes to 80% A nucleotides and 20% G nucleotides (consistent with the repeat motif AAAAG) for ∼150 bp. The consensus sequences displayed in Fig. 4 are derived from these plots.

**Figure 4:**
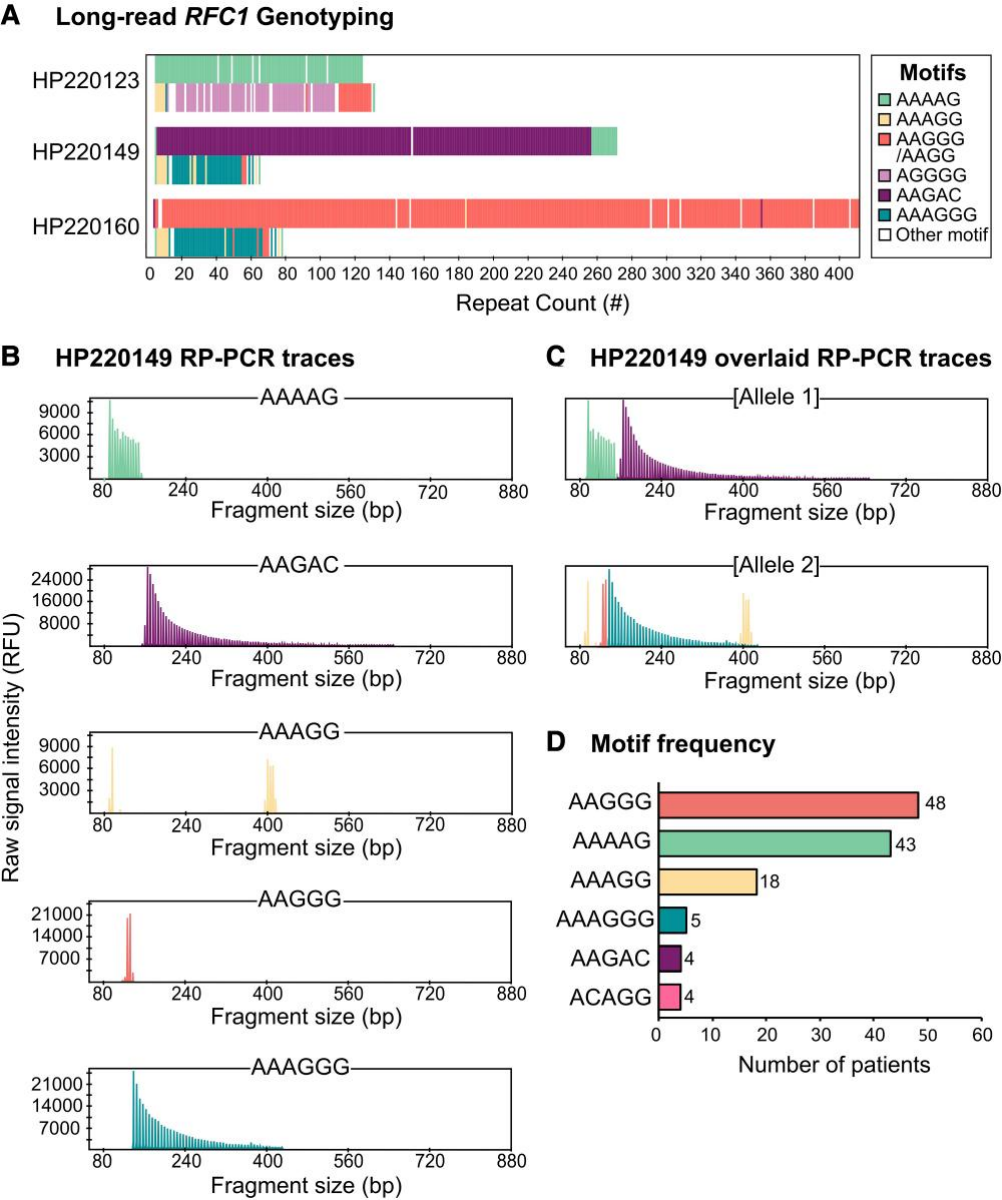
**Additional repeat motifs detected by targeted long-read sequencing.** (**A**) Consensus sequences generated from long-read sequencing of three patient samples. (**B**) Traces for each motif-specific RP-PCR for patient sample HP220149. (**C**) Shows the separate motif-specific RP-PCR traces overlayed to correspond with the alleles determined by long-read sequencing for sample HP220149. (**D**) Plot displaying the number of times each repeat motif was detected within samples harbouring biallelic expansions (*n* = 82) as determined by RP-PCR.

The error rate of ONT long-read sequencing means that there is some ambiguity in determining the exact repeat present for certain alleles (Fig. 3). Where possible, we employed repeat motif-specific RP-PCR to confirm the composition of the motif present. The consensus sequences are displayed in Fig. 4A.

Sample HP220149 initially showed a few peaks on each of the AAAAG, AAAGG and AAGGG RP-PCR traces with large gaps between these peaks (Fig. 4B). Long-read sequencing uncovered the presence of two novel motifs that constituted the major proportion of each allele. Allele 1 was determined to contain ∼270 repeats consisting primarily of the novel motif AAGAC, with a small 5ʹ segment of the motif AAAAG which had previously been captured by RP-PCR. Allele 2 was determined to contain ∼60 repeats, consisting primarily of the novel hexanucleotide motif AAAGGG, with small segments of AAAGG and AAGGG repeats that had also been captured in the RP-PCR data (Fig. 4). Specific RP-PCR assays, devised for novel repeats AAGAC and AAAGGG, confirmed their presence within the sample, and demonstrate the concordance between the RP-PCR assays and long-read sequencing.

The RP-PCR data for sample HP220160 indicated that one allele contained an expansion of the pathogenic AAGGG repeat. Peaks shown on the AAAGG repeat trace were separated by a substantial gap, indicating the presence of a second allele that contains an unknown repeat motif. The long-read sequencing results confirmed that Allele 1 contained a pure AAGGG repeat expansion of ∼400 repeats. Allele 2 was shown to be expanded to ∼70 repeats and contained the same AAAGGG hexanucleotide repeat, seen in HP220149, constituting the majority of the allele. Interestingly, the overall composition of the second allele was very similar to that of HP220149, with the same combination of motifs (AAAGG, AAAGGG and AAGGG) present in similar locations for both samples (Fig. 4A). These motifs were also confirmed by RP-PCR.

When first tested by RP-PCR, sample HP220123 indicated the presence of a pure AAAAG expansion on one allele and showed multiple peaks presented on the AAGGG repeat trace as a portion of the second allele. Long-read sequencing confirmed the presence of a pure AAAAG expansion of ∼125 repeats within Allele 1 and uncovered one novel motif present within Allele 2. Allele 2 was determined to contain ∼130 repeats and consisted of the AAGGG repeat motif followed by the motif AGGGG (Fig. 4A). The high proportion of G nucleotides present in conjunction with the intrinsic indel error rate of ONT sequencing makes it difficult to discern the exact repeat motif distribution. RP-PCR assays for AGGGG failed, possibly due to the high GC content and the likely impure nature of the expansion. This meant that we were unable to confirm the exact motif present by RP-PCR.

Following validation of RP-PCR assays developed for AAGAC and AAAGGG, all samples with biallelic expansions indicated by flanking PCR were tested to determine the prevalence of these motifs within the cohort. As displayed in Fig. 4, the RP-PCRs show concordance with the results generated by targeted long-read sequencing. Figure 4D shows the prevalence of each repeat motif tested by RP-PCR within this cohort of 82 samples. Motifs were recorded to be present within patient samples that showed ≥10 continuous peaks by repeat-specific RP-PCR (as phasing alleles is not possible by RP-PCR, prevalence is measured in terms of patient samples rather than alleles). As anticipated, the most common motifs were AAGGG, AAAAG and AAAGG, with repeats detected in 58.5% (*n* = 48), 52.4% (*n* = 43) and 22.0% (*n* = 18) of patients harbouring biallelic expansions, respectively. The ACAGG repeat motif was observed in 4.9% (*n* = 4) of patients. The prevalence of the newly described repeat motifs was 6.1% for AAAGGG (*n* = 5) and 4.9% for AAGAC (*n* = 4).

### Investigation of *RFC1* repeat motifs in gnomAD

To further explore the extent of heterogeneity displayed at the *RFC1* locus, we interrogated 19 241 genomes from gnomAD v3.^27^ In addition to the reference AAAAG, a total of 19 unique repeat motifs were identified, including 15 that have not been described previously in the literature. The different repeat motifs and corresponding frequencies are displayed in Table 1. As shown in Table 1, all three of the motifs observed by targeted long-read sequencing were detected within the 19 241 genomes at low frequencies.

**Table 1:** Alternate repeat motifs at the *RFC1* locus seen in 19 241 genomes

Frequency in 19 241 genomes	Repeat motif
*1202*	AAGGG
*1914*	AAAGG
*7*	ACAGG
*474*	AAGAG
*2*	AAAAA
*1*	AAAAC
*1*	AAACG
*6*	AACAG
*59*	AAGAC
*9*	AACGG
*7*	AAGGC
*7*	AAGGT
*2*	ACGGG
*5*	AGGGC
*2*	AGGGG
*2*	AGGTG
*9*	AAAAAG
*2*	AAAAGG
*3*	AAAGGG

Analysis of short-read whole-genome sequencing (sr-WGS) data provides a gauge for the frequency of different motifs. However, it is limited to the detection of motifs close to the flanking regions of the repeat; therefore, motifs that occur as part of complex alleles further into the expansion may not have been detected through these methods, affecting the measurement of their overall prevalence within the 19 241 genomes.

## Discussion

### *RFC1* biallelic pathogenic expansions are prevalent within Australasian neurological disease patients

Within this study, we identified biallelic pathogenic *RFC1* expansions in 15.3% of our cohort of 242 Australasian, neurological disease patients. With 3.8% of ataxia patients, 8% of neuropathy patients, 31.1% of ataxia with neuropathy patients and 35.7% of clinically suspected patients with CANVAS harbouring biallelic *RFC1* expansions.

The prevalence of biallelic *RFC1* pathogenic expansions has been shown to vary significantly between different late-onset ataxia cohorts, ranging from 1.1% in a Canadian-Brazilian study^6^ to 28.9% in a British cohort study^13^ (3.2% in a North American cohort,^30^ 6.5% within a Greek cohort,^31^ 5.2–10.8% within Japanese cohorts,^8,10^ 14% in a Turkish cohort,^32^ 14.5% within an Italian cohort,^33^ 15% in a French cohort^34^ and 20.2% within a German cohort^35^). The results of our study fall within this range.

Certain pathogenic motifs appear to be enriched in different populations. Initial studies associating the AAGGG pathogenic expansion with disease described a core haplotype and postulated a single origin of the expanded allele, indicating that its prevalence may be highest in European, particularly British, populations.^4,5^ The AAGGG expansion occurs at a similar allele frequency in Asian populations, although the contribution of *RFC1* to disease in these populations remains much lower compared with European studies. Interestingly, a number of population-based studies reported higher carrier frequencies of the pathogenic AAGGG motif in conjunction with lower biallelic rates.^6,10,36^ This indicates that discrepancies in selection criteria are likely to account for a large proportion of the variability in overall prevalence seen across the numerous cohort studies focusing on late-onset ataxias. Another possibility might be population-specific modifying factors. Alternatively, there may be a more prominent unknown cause of ataxia/CANVAS within these populations that is overshadowing *RFC1*-positive CANVAS samples.

Two additional pathogenic alleles have since been described: denoted the Māori allele, an allele comprising both expanded AAAGG and AAGGG motifs, was originally observed to be specific to the Māori populations of New Zealand and the Cook Islands, it has recently also been reported in a Japanese patient.^9,37^ The ACAGG repeat motif has been reported in patients of Niue, Chinese and Japanese origin, indicating that it is likely to be prevalent in East Asian populations.^7,8^ As Australasia consists of Australia, New Zealand, New Guinea and some of the small neighbouring islands, there is representation of each of the different pathogenic alleles within our cohort.

The inclusion criteria for this cohort were intentionally broad and relied primarily on the often limited clinical details provided at the time of request of initial diagnostic genetic testing and an age cut-off of >30 years, as CANVAS has generally been described as a late-onset disease. Thus, we predict that the diagnostic yield obtained here would be reflective of the yields seen in other diagnostic laboratories within Australia and countries with similar demographics.

CANVAS is a syndrome that is characterized by a triad of key features: cerebellar ataxia, sensory neuropathy/neuronopathy and vestibular involvement.^1‐3,13^ Within our study diagnostic success rate for pathogenic *RFC1* expansions was shown to increase from 3.8 and 8% in patients referred as only manifesting one of the characteristic features (ataxia or neuropathy), to 31.1% in patients exhibiting two core features (ataxia and neuropathy) and to 35.7% in patients with clinically suspected CANVAS. This trend is concordant with previous studies and strengthens the notion that variable disease progression contributes to the phenotypic heterogeneity observed within *RFC1*-related disease. However, the limited access to formal vestibular testing in particular may have underestimated the number of patients who had vestibular involvement.

Though many cohorts have assessed the prevalence of *RFC1* pathogenic expansions within late-onset ataxia cohorts, fewer have screened neuropathy-only cohorts. One study reported that 34% of sensory neuropathy patients had pathogenic biallelic *RFC1* expansions of which 42% exhibited sensory neuropathy only.^14^ Our results confirm the importance of screening for *RFC1* expansions with a similar prevalence among neuropathy-only patients as among those manifesting ataxia without neuropathy. As expected, the diagnostic yields were lower for patients with unsolved isolated ataxias or isolated neuropathies, as these phenotypes encompass a wider range of disease aetiologies. However, these patients contribute to a greater total number of diagnoses than patients with clinically suspected CANVAS. Therefore, screening these cohorts would still yield a significant number of *RFC1* diagnoses.

Here, we also demonstrate how screening for the biallelic expansions within the *RFC1* gene can be incorporated into targeted gene panels using NGS. This allows for all ataxia and neuropathy samples that are tested on the targeted gene panels to be concurrently screened for the *RFC1* expansion, without additional testing being conducted on the sample. This is especially helpful for patients with fewer differential features indicative of CANVAS, who would not be routinely tested for *RFC1* expansions. This would also encompass recent phenotype expansions such as multiple system atrophy and Parkinsonism and provides a catch-all approach without onerous testing.^16,18‐20^

### Increased heterogeneity at the *RFC1* locus

Seven different expanded alleles have already been described at the *RFC1* disease locus. Three that have been associated with diseases: AAGGG, ACAGG and the Māori allele [(AAAGG)_15–25_(AAGGG)_exp_(AAAGG)_10_] and four that are observed to be benign or of undetermined pathogenicity: AAAAG, AAAGG, AAGAG and AGAGG.^4‐7,9^

Here, we add to what has already been shown to be a genetically heterogeneous locus. Within the 82 samples that were tested by RP-PCR, a large proportion contained gaps within the resulting traces. This prompted the implementation of targeted long-read sequencing through which we uncovered an additional three repeat motifs; two pentanucleotide as well as one hexanucleotide repeat: AAGAC, AGGGG and AAAGGG.

In addition, we observed many complex alleles constituted of multiple repeat motifs, as seen in the Māori allele, further extending the variability seen at the locus. In fact, the majority of samples harbouring biallelic expansions were indicated to have at least one complex allele. Interruptions, defined as one to nine continuous peaks on RP-PCR, were also apparent within many alleles.

Both AAGAC and AAAGGG motifs were shown to be recurrent within the cohort and were observed in four and five individuals, respectively, by RP-PCR. This indicates that these motifs are reasonably common. Overall, 11% of patients who had biallelic expansions, within our cohort, had expanded alleles that contained a repeat motif that had not been previously reported. Interrogation of 19 241 whole genomes from the gnomAD data set confirmed that AAGAC, AAAGGG and AGGGG were present within the population at low frequencies.^27^ An additional 12 repeat motifs, that had not previously been reported, were also detected to be present at the *RFC1* locus within the gnomAD data set. Between the sr-WGS data set and the samples screened within our cohort, a total of 19 motifs, alternate to the reference AAAAG, were observed at the *RFC1* locus.

The prevalence of the AAGAC and AAAGGG motifs was significantly lower within the gnomAD data set in comparison with our cohort. These motifs were often part of complex alleles with a few repeats of an alternate repeat motif on the flanking ends. Though largely improved, STR analysis conducted on sr-WGS data is limited. Genomic regions that are highly repetitive or GC rich are not captured well during sequencing. Therefore, large expansions or GC-rich motifs at the *RFC1* locus may result in poor sequencing coverage.^21,38,39^ Additionally, reads containing novel repeat motifs, particularly those without flanking segments (or paired with flanking reads) may not align to the correct locus. Therefore, novel motifs containing higher GC content or occurring further into the repeat expansion may be missed on sr-WGS.

### Association of novel repeat motifs with disease

Interpreting the pathogenicity of the novel repeat motifs reported here is challenging. There appears to be an association between GC content and the size of expansion with pathogenicity of an allele.^4,7^ The AGGGG motif contains a higher GC content (80%) than any previously reported configuration. AAGAC has a lower GC content similar to that of the AAAGG benign allele, whereas the AAAGGG repeat has 50% GC content, slightly less than reported pathogenic alleles. However, the size of these expanded alleles (∼60–270 repeats) is more reflective of the previously described benign AAAAG configuration (∼15–200 repeats).^4^ The key difference here is that all the configurations were observed as part of complex alleles, containing segments of different repeat motifs and interruptions throughout. The stabilizing effects of interruptions are common within other repeat expansion disorders.^40^ The impure expansions observed here may experience a similar stabilizing effect preventing the repeats from expanding to larger sizes.

Though theorized, *RFC1* expansion size has not been definitively linked with disease causation. It is possible that pathogenicity is more reliant on the specific repeat configuration present than on expansion size. CANVAS is not the only disease associated with an expansion of a repeat configuration that is different from the reference sequence. Both spinocerebellar ataxia type 37 and the suite of adult familial myoclonic epilepsies (FAMEs 1, 2, 3, 4, 6, 7) have non-reference expansions associated with disease.^41‐47^ Interestingly, both diseases have the same reference motif (ATTTT) and expanded pathogenic motif (ATTTC) within their respective genes; *DAB1* and *SAMD12*, *STARD7*, *MARCH6*, *YEATS2*, *TNRC6A* and *RAPGEF2*. The *SAMD12* locus was also shown to exhibit additional heterogeneity with the report of a novel repeat motif expansion (ATTTG) inserted at the same locus, shown to segregate with disease in a Chinese family with FAME.^48^ Functional studies of the *DAB1* gene have shown that pathogenicity is repeat motif dependent. Overexpression of alleles with the ATTTC insertion was shown to cause RNA aggregate formation, whereas overexpression of the expanded ATTTT allele did not.^49^ The RNA aggregates were observed to localize in the nucleus, resulting in neurotoxicity.^49^ Similar pathomechanisms have been suggested for the FAME loci.

Understanding the pathomechanism underlying *RFC1*-related diseases is vital for interpreting the effect of these novel repeat motifs, interruptions and complex alleles. Without this mechanism, the field is reliant on deep phenotyping studies which may not be feasible with rarer motifs.

### Implications for diagnostic service providers

The increased heterogeneity observed adds to the intrinsic limitations of RP-PCR for the diagnosis of CANVAS. RP-PCR is unable to phase expanded alleles and therefore relies on the assumption that there are no other untested benign expansions present when one observes apparent homozygosity for a particular motif. This is particularly problematic given the number of previously unreported motifs observed within our study. Additionally, RP-PCR only provides insight into the configuration of the first ∼160 repeats of the expansions at most. Given that pathogenic expansions often reach over 1000 repeats, the majority of the expanded allele is inferred, and any interruptions or changes in motif past this detection limit will go undetected. Given the substantial variability and complexity of expanded alleles we have shown in our cohort, this appears to be a major caveat to diagnostics relying on RP-PCR alone.

As shown here, targeted long-read sequencing has the ability to fully capture the entire expanded *RFC1* allele, as well as phase the alleles, providing an accurate and definitive genotype. Implementation of targeted long-read sequencing for *RFC1* diagnostics will allow patients with homozygous pathogenic alleles to be confidently diagnosed. For cases containing novel repeat motifs or alleles for which the pathogenicity has not been determined, further studies will be required.

The recent description of CANVAS associated with loss of function (LoF) SNVs in conjunction with an expanded AAGGG allele in *RFC1* adds to the genetic complexity of this disease and further complicates diagnostic testing.^11,12^ Future studies will need to evaluate the proportion of patients with single pathogenic expansions that harbour a LoF variant in trans.

**Figure 5:**
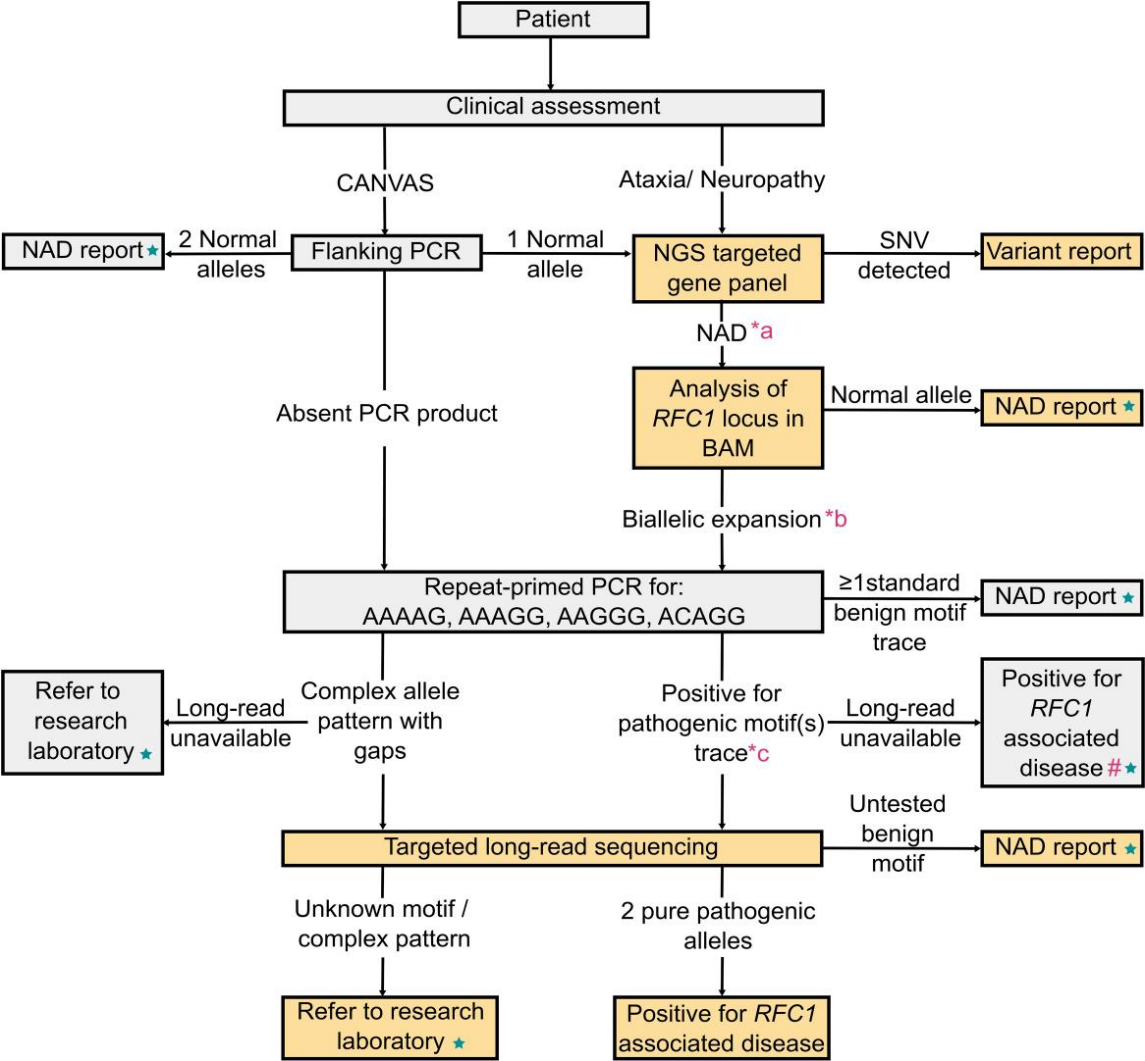
**Suggested diagnostic workflow for the detection of biallelic pathogenic *RFC1* expansions.** Incorporation of probes spanning all exons of *RFC1*, in addition to the repeat region, will allow concurrent screening for recently discovered LoF variants, where targeted sequencing is available. Star symbols indicate points at which we recommend testing for additional repeat expansion disorders, where available, to exclude alternative causes or as cascade testing following a negative *RFC1* result. Additional interpretation criteria are denoted by the asterisk and hash symbols: (*a) or *RFC1* LoF variant (*b) or *RFC1* LoF variant in combination with a single expanded allele (*c) or one pure pathogenic expansion in combination with *RFC1* LoF variant (#) include the limitations of testing on the report. NAD, no abnormalities detected.

In Fig. 5, we suggest a workflow for diagnostic laboratories that can be adapted to fit the needs and resources of different laboratories. The workflow outlines the current PCR-based methods and incorporates NGS and long-read sequencing to improve diagnostic rates and overcome the limitations of RP-PCR to provide a more accurate genetic diagnosis for patients.

As costs of sequencing continue to drop, the RP-PCR step could be skipped to further streamline the process. In laboratories where long-read sequencing is not feasible due to resource availability or financial constraints, testing using the combination or flanking PCR and RP-PCR for the most common benign motifs within the population (AAAAG and AAAGG) as well as the known pathogenic motifs (AAGGG and ACAGG) is adequate, provided the limitations of testing are emphasized when reported. The recent traction of Bionano optical mapping provides an alternative method, which may be easier to implement diagnostically in present times. It can facilitate accurate sizing and phasing of expanded alleles, which can aid in determining pathogenicity. However, the technology is not capable of determining the repeat motif or the presence of any interruptions within the expanded region. In the case of *RFC1*, given the complexities noted, we believe integration of targeted long-read sequencing technologies into diagnostic laboratories will be essential to facilitate accurate diagnoses of patients.

## Conclusion

Pathogenic biallelic expansions in *RFC1* underlie a significant proportion (15.3%) of unsolved ataxia and neuropathy patients in Australasia, affirming the need for accessible and comprehensive *RFC1* diagnostic testing. The *RFC1* locus was shown to be far more heterogeneous and complex than previously described. Within this cohort we uncovered three novel repeat motifs: AAGAC, AAAGGG, AGGGG, and observed a high prevalence of expansions consisting of more than a single repeat motif. Furthermore, the computational analysis of 19 241 genomes from gnomAD revealed a total of 19 motifs present at the *RFC1* locus that differed from the reference. Standard genomic diagnostic approaches, such as RP-PCR, are unable to fully capture and elucidate the complexity of the locus. Our study showcases the capabilities of NGS targeted sequencing as a screening tool and highlights the importance of integrating long-read sequencing into the diagnostic space to overcome current limitations.

## Supplementary Material

fcad208_Supplementary_Data

## Data Availability

The data that support the findings of this study are available from the corresponding author upon request.

## References

[1] Migliaccio AA , Halmagyi GM, McGarvie LA, Cremer PD. Cerebellar ataxia with bilateral vestibulopathy: Description of a syndrome and its characteristic clinical sign. Brain. 2004;127(Pt 2):280–293. 14607788 10.1093/brain/awh030

[2] Szmulewicz DJ , Waterston JA, MacDougall HG, et al Cerebellar ataxia, neuropathy, vestibular areflexia syndrome (CANVAS): A review of the clinical features and video-oculographic diagnosis. Ann NY Acad Sci. 2011;1233:139–147. 21950986 10.1111/j.1749-6632.2011.06158.x

[3] Szmulewicz DJ , Merchant SN, Halmagyi GM. Cerebellar ataxia with neuropathy and bilateral vestibular areflexia syndrome: A histopathologic case report. Otol Neurotol. 2011;32(8):E63–E65. 21451431 10.1097/MAO.0b013e318210b719 PMC3128688

[4] Cortese A , Simone R, Sullivan R, et al Biallelic expansion of an intronic repeat in RFC1 is a common cause of late-onset ataxia. Nat Genet. 2019;51(4):649–658. 30926972 10.1038/s41588-019-0372-4 PMC6709527

[5] Rafehi H , Szmulewicz DJ, Bennett MF, et al Bioinformaticsbased identification of expanded repeats: A non-reference intronic pentamer expansion in RFC1 causes CANVAS. Am J Hum Genet. 2019;105(1):151–165. 31230722 10.1016/j.ajhg.2019.05.016 PMC6612533

[6] Akçimen F , Ross JP, Bourassa CV, et al Investigation of the RFC1 repeat expansion in a Canadian and a Brazilian ataxia cohort: Identification of novel conformations. Front Genet. 2019;10:1219. 31824583 10.3389/fgene.2019.01219 PMC6884024

[7] Scriba CK , Beecroft SJ, Clayton JS, et al A novel RFC1 repeat motif (ACAGG) in two Asia-Pacific CANVAS families. Brain. 2020;143(10):2904–2910. 33103729 10.1093/brain/awaa263 PMC7780484

[8] Tsuchiya M , Nan H, Koh K, et al RFC1 repeat expansion in Japanese patients with late-onset cerebellar ataxia. J Hum Genet. 2020;65(12):1143–1147. 32694621 10.1038/s10038-020-0807-x

[9] Beecroft SJ , Cortese A, Sullivan R, et al A Maori specific RFC1 pathogenic repeat configuration in CANVAS, likely due to a founder allele. Brain. 2020;143(9):2673–2680. 32851396 10.1093/brain/awaa203 PMC7526724

[10] Miyatake S , Yoshida K, Koshimizu E, et al Repeat conformation heterogeneity in cerebellar ataxia, neuropathy, vestibular areflexia syndrome. Brain. 2022;145(3):1139–1150. 35355059 10.1093/brain/awab363

[11] Benkirane M , Da Cunha D, Marelli C, et al RFC1 nonsense and frameshift variants cause CANVAS: Clues for an unsolved pathophysiology. Brain. 2022;145:3770–3775. 35883251 10.1093/brain/awac280

[12] Ronco R , Perini C, Curro R, et al Truncating variants in RFC1 in cerebellar ataxia, neuropathy, and vestibular areflexia syndrome. Neurology. 2022;100:e543–e554. 36289003 10.1212/WNL.0000000000201486 PMC9931080

[13] Cortese A , Tozza S, Yau WY, et al Cerebellar ataxia, neuropathy, vestibular areflexia syndrome due to RFC1 repeat expansion. Brain. 2020;143(2):480–490. 32040566 10.1093/brain/awz418 PMC7009469

[14] Currò R , Salvalaggio A, Tozza S, et al RFC1 expansions are a common cause of idiopathic sensory neuropathy. Brain. 2021;144(5):1542–1550. 33969391 10.1093/brain/awab072 PMC8262986

[15] Kumar KR , Cortese A, Tomlinson SE, et al RFC1 expansions can mimic hereditary sensory neuropathy with cough and Sjogren syndrome. Brain. 2020;143(10):e82. 32949124 10.1093/brain/awaa244 PMC7586083

[16] Herrmann L , Gelderblom M, Bester M, et al Multisystemic neurodegeneration caused by biallelic pentanucleotide expansions in RFC1. Parkinsonism Relat Disord. 2022;95:54–56. 35030450 10.1016/j.parkreldis.2022.01.001

[17] Colucci F , Di Bella D, Pisciotta C, et al Beyond canvas: Behavioral onset of rfc1-expansion disease in an Italian family-causal or casual? Neurol Sci. 2022;43(8):5095–5098. 35585435 10.1007/s10072-022-06137-1

[18] da Silva Schmitt G , Martinez ARM, da Graca FF, et al Dopa-responsive parkinsonism in a patient with homozygous RFC1 expansions. Mov Disord. 2020;35(10):1889–1890. 33068476 10.1002/mds.28286

[19] Sullivan R , Yau WY, Chelban V, et al RFC1-related ataxia is a mimic of early multiple system atrophy. J Neurol Neurosurg Psychiatry. 2021;92:444–446. 33563805 10.1136/jnnp-2020-325092 PMC7958109

[20] Tagliapietra M , Cardellini D, Ferrarini M, et al RFC1 AAGGG repeat expansion masquerading as chronic idiopathic axonal polyneuropathy. J Neurol. 2021;268(11):4280–4290. 33884451 10.1007/s00415-021-10552-3 PMC8505379

[21] Chintalaphani SR , Pineda SS, Deveson IW, Kumar KR. An update on the neurological short tandem repeat expansion disorders and the emergence of long-read sequencing diagnostics. Acta Neuropathol Commun. 2021;9(1):98. 34034831 10.1186/s40478-021-01201-x PMC8145836

[22] Jain M , Koren S, Miga KH, et al Nanopore sequencing and assembly of a human genome with ultra-long reads. Nat Biotechnol. 2018;36(4):338–345. 29431738 10.1038/nbt.4060 PMC5889714

[23] Wenger AM , Peluso P, Rowell WJ, et al Accurate circular consensus long-read sequencing improves variant detection and assembly of a human genome. Nat Biotechnol. 2019;37(10):1155–1162. 31406327 10.1038/s41587-019-0217-9 PMC6776680

[24] Stevanovski I , Chintalaphani SR, Gamaarachchi H, et al Comprehensive genetic diagnosis of tandem repeat expansion disorders with programmable targeted nanopore sequencing. Sci Adv. 2022;8(9):eabm5386. 35245110 10.1126/sciadv.abm5386 PMC8896783

[25] Nakamura H , Doi H, Mitsuhashi S, et al Long-read sequencing identifies the pathogenic nucleotide repeat expansion in RFC1 in a Japanese case of CANVAS. J Hum Genet. 2020;65(5):475–480. 32066831 10.1038/s10038-020-0733-y

[26] Beecroft SJ , Yau KS, Allcock RJN, et al Targeted gene panel use in 2249 neuromuscular patients: The Australasian referral center experience. Ann Clin Transl Neurol. 2020;7(3):353–362. 32153140 10.1002/acn3.51002 PMC7086001

[27] Karczewski KJ , Francioli LC, Tiao G, et al The mutational constraint spectrum quantified from variation in 141,456 humans. Nature. 2020;581(7809):434–443. 32461654 10.1038/s41586-020-2308-7 PMC7334197

[28] Dolzhenko E , Deshpande V, Schlesinger F, et al ExpansionHunter: A sequence-graph-based tool to analyze variation in short tandem repeat regions. Bioinformatics. 2019;35(22):4754–4756. 31134279 10.1093/bioinformatics/btz431 PMC6853681

[29] Dolzhenko E , Weisburd B, Ibañez K, et al REViewer: Haplotype-resolved visualization of read alignments in and around tandem repeats. Genome Med. 2022;14(1):84. 35948990 10.1186/s13073-022-01085-z PMC9367089

[30] Aboud Syriani D , Wong D, Andani S, et al Prevalence of RFC1-mediated spinocerebellar ataxia in a North American ataxia cohort. Neurol Genet. 2020;6(3):e440. 32582864 10.1212/NXG.0000000000000440 PMC7274910

[31] Kontogeorgiou Z , Kartanou C, Tsirligkani C, et al Biallelic RFC1 pentanucleotide repeat expansions in Greek patients with late-onset ataxia. Clin Genet. 2021;100(1):90–94. 33745133 10.1111/cge.13960

[32] Traschutz A , Cortese A, Reich S, et al Natural history, phenotypic spectrum, and discriminative features of multisystemic RFC1 disease. Neurology. 2021;96(9):e1369–e1382. 33495376 10.1212/WNL.0000000000011528 PMC8055326

[33] Barghigiani M , De Michele G, Tessa A, et al Screening for RFC-1 pathological expansion in late-onset ataxias: A contribution to the differential diagnosis. J Neurol. 2022;269:5431–5435. 35633373 10.1007/s00415-022-11192-x

[34] Montaut S , Diedhiou N, Fahrer P, et al Biallelic RFC1-expansion in a French multicentric sporadic ataxia cohort. J Neurol. 2021;268(9):3337–3343. 33666721 10.1007/s00415-021-10499-5

[35] Gisatulin M , Dobricic V, Zuhlke C, et al Clinical spectrum of the pentanucleotide repeat expansion in the RFC1 gene in ataxia syndromes. Neurology. 2020;95(21):e2912–e2923. 32873692 10.1212/WNL.0000000000010744

[36] Fan Y , Zhang S, Yang J, et al No biallelic intronic AAGGG repeat expansion in RFC1 was found in patients with late-onset ataxia and MSA. Parkinsonism Relat Disord. 2020;73:1–2. 32151945 10.1016/j.parkreldis.2020.02.017

[37] Ando M , Higuchi Y, Yuan JH, et al Genetic and clinical features of cerebellar ataxia with RFC1 biallelic repeat expansions in Japan. Front Neurol. 2022;13:952493. 36034314 10.3389/fneur.2022.952493 PMC9404689

[38] Lelieveld SH , Spielmann M, Mundlos S, Veltman JA, Gilissen C. Comparison of exome and genome sequencing technologies for the complete capture of protein-coding regions. Hum Mutat. 2015;36(8):815–822. 25973577 10.1002/humu.22813 PMC4755152

[39] Ebbert MTW , Jensen TD, Jansen-West K, et al Systematic analysis of dark and camouflaged genes reveals disease-relevant genes hiding in plain sight. Genome Biol. 2019;20(1):97. 31104630 10.1186/s13059-019-1707-2 PMC6526621

[40] Khristich AN , Mirkin SM. On the wrong DNA track: Molecular mechanisms of repeat-mediated genome instability. J Biol Chem. 2020;295(13):4134–4170. 32060097 10.1074/jbc.REV119.007678 PMC7105313

[41] Corral-Juan M , Serrano-Munuera C, Rabano A, et al Clinical, genetic and neuropathological characterization of spinocerebellar ataxia type 37. Brain. 2018;141(7):1981–1997. 29939198 10.1093/brain/awy137

[42] Ishiura H , Doi K, Mitsui J, et al Expansions of intronic TTTCA and TTTTA repeats in benign adult familial myoclonic epilepsy. Nat Genet. 2018;50(4):581. 29507423 10.1038/s41588-018-0067-2

[43] Cen Z , Jiang Z, Chen Y, et al Intronic pentanucleotide TTTCA repeat insertion in the SAMD12 gene causes familial cortical myoclonic tremor with epilepsy type 1. Brain. 2018;141(8):2280–2288. 29939203 10.1093/brain/awy160

[44] Corbett MA , Kroes T, Veneziano L, et al Intronic ATTTC repeat expansions in STARD7 in familial adult myoclonic epilepsy linked to chromosome 2. Nat Commun. 2019;10(1):4920. 31664034 10.1038/s41467-019-12671-y PMC6820779

[45] Florian RT , Kraft F, Leitão E, et al Unstable TTTTA/TTTCA expansions in MARCH6 are associated with familial adult myoclonic epilepsy type 3. Nat Commun. 2019;10(1):4919. 31664039 10.1038/s41467-019-12763-9 PMC6820781

[46] Yeetong P , Pongpanich M, Srichomthong C, et al TTTCA repeat insertions in an intron of YEATS2 in benign adult familial myoclonic epilepsy type 4. Brain. 2019;142(11):3360–3366. 31539032 10.1093/brain/awz267

[47] Lei XX , Liu Q, Lu Q, et al TTTCA repeat expansion causes familial cortical myoclonic tremor with epilepsy. Eur J Neurol. 2019;26(3):513–518. 30351492 10.1111/ene.13848

[48] Cen Z , Chen Y, Yang D, et al Introsnic (TTTGA)n insertion in SAMD12 also causes familial cortical myoclonic tremor with epilepsy. Mov Disord. 2019;34(10):1571–1576. 31483537 10.1002/mds.27832

[49] Seixas AI , Loureiro JR, Costa C, et al A pentanucleotide ATTTC repeat insertion in the non-coding region of DAB1, mapping to SCA37, causes spinocerebellar ataxia. Am J Hum Genet. 2017;101(1):87–103. 28686858 10.1016/j.ajhg.2017.06.007 PMC5501871

