# Supplementary material for: RFC1 in an Australasian neurological disease cohort: extending the genetic heterogeneity and implications for diagnostics
Source: Brain Commun. 2023 Jul 25;5(4):fcad208. doi: 10.1093/braincomms/fcad208 (PMC10445415; doi:10.1093/braincomms/fcad208)
Supplement: fcad208_Supplementary_Data [file fcad208_supplementary_data.pdf]

<table>
<tr><th>Protocol</th><th>Primers (5’-3’)</th><th>Cycling conditions</th><th>Reaction components</th></tr>
<tr><td>Flanking PCR</td><td>Forward:
TCAAGTGATACTCCAGCTACACC GTTGC

Reverse:
CAGCATTGTGGGAGACAGGCCA ATCACT</td><td><table><tr><th>Temp (˚C)</th><th>Time</th><th>Cycles</th></tr><tr><td>95</td><td>5m</td><td></td></tr><tr><td>95</td><td>30s</td><td rowspan="3">35</td></tr><tr><td>65</td><td>30s</td></tr><tr><td>72</td><td>30s</td></tr><tr><td>4</td><td>hold</td><td></td></tr></table></td><td>20 µl reaction
1% HotStarTaq DNA Polymerase
10% HotStarTaq 10X PCR Buffer (15mM Mg)
0.2 µM forward primer
0.2 µM reverse primer 5% DMSO
50 ng DNA
Nuclease Free water to 20 µl</td></tr>
<tr><td colspan="4">Repeat-primed PCRs</td></tr>
<tr><td>AAAAG RP-PCR</td><td>P1: famTCAAGTGATACTCCAGCTAC ACCGT
P3: CAGGAAACAGCTATGACC
P4: CAGGAAACAGCTATGACCGAAAA GAAAAGAAAAGAAAA</td><td><table><tr><th>Temp (˚C)</th><th>Time</th><th>Cycles</th></tr><tr><td>98</td><td>3m</td><td></td></tr><tr><td>98</td><td>10s</td><td rowspan="3">35</td></tr><tr><td>55</td><td>15s</td></tr><tr><td>72</td><td>2m</td></tr><tr><td>72</td><td>1m</td><td></td></tr><tr><td>4</td><td>hold</td><td></td></tr></table></td><td rowspan="6">15 µl reaction
7.5 µl (1X) 2X Phusion Flash PCR Master Mix (Thermo-Fisher)
3% DMSO
0.5 µM FAM forward Primer (P1)
0.5 µM Anchor Primer (P2)
0.05 µM Configuration specific reverse Primer (P4)
50 ng DNA
Nuclease Free water to 15 µl</td></tr>
<tr><td>AAAGG RP-PCR</td><td>P1: famTCAAGTGATACTCCAGCTAC ACCGT
P3: CAGGAAACAGCTATGACC
P4: CAGGAAACAGCTATGACCGGAAA GGAAAGGAAAGGAAA</td><td rowspan="3"><table><tr><th>Temp (˚C)</th><th>Time</th><th>Cycles</th></tr><tr><td>98</td><td>3m</td><td></td></tr><tr><td>98</td><td>10s</td><td rowspan="3">35</td></tr><tr><td>65</td><td>15s</td></tr><tr><td>72</td><td>2m</td></tr><tr><td>72</td><td>1m</td><td></td></tr><tr><td>4</td><td>hold</td><td></td></tr></table></td></tr>
<tr><td>AAGAC RP-PCR</td><td>P1: famTCAAGTGATACTCCAGCTAC ACCGT
P3: CAGGAAACAGCTATGACC
P4: CAGGAAACAGCTATGACCAAGACA AGACAAGACAAGAC</td></tr>
<tr><td>AAAGGG RP-PCR</td><td>P1: famTCAAGTGATACTCCAGCTAC ACCGT
P3: CAGGAAACAGCTATGACC
P4: CAGGAAACAGCTATGACCAAA GGGAAAGGGAAAGGG</td></tr>
<tr><td>AAGGG RP-PCR</td><td>P1: famTCAAGTGATACTCCAGCTAC ACCGT
P3: CAGGAAACAGCTATGACC
P4:CAGGAAACAGCTATGACCGGGAA GGGAAGGGAAGGGAA</td><td rowspan="2"><table><tr><th>Temp (˚C)</th><th>Time</th><th>Cycles</th></tr><tr><td>98</td><td>3m</td><td></td></tr><tr><td>98</td><td>10s</td><td rowspan="3">35</td></tr><tr><td>70</td><td>15s</td></tr><tr><td>72</td><td>2m</td></tr><tr><td>72</td><td>1m</td><td></td></tr><tr><td>4</td><td>hold</td><td></td></tr></table></td></tr>
<tr><td>ACAGG RP-PCR</td><td>P1: famTCAAGTGATACTCCAGCTAC ACCGT
P3: CAGGAAACAGCTATGACC
P4: CAGGAAACAGCTATGACCACAGG ACAGGACAGGACAGG</td></tr>
</table>

Supplementary table 1| Details of PCR protocols. Provides specifications of reaction composition, thermocycling conditions and the primer details for the PCR-based assays used for characterisation of the *RFC1* repeat expansions including flanking PCR and repeat-primed PCR for AAAAG, AAAGG, AAGGG, ACAGG, AAGAC and AAAGGG.
